# Crystal Chemistry and Physics of UCd_11_

**DOI:** 10.1021/acs.inorgchem.2c01986

**Published:** 2022-11-29

**Authors:** Nazar Zaremba, Kristian Witthaut, Yurii Prots, Mitja Krnel, Ulrich Burkhardt, Zachary Fisk, Yuri Grin, Eteri Svanidze

**Affiliations:** † Department Chemische Metallkunde, Max-Planck-Institut für Chemische Physik fester Stoffe, Nöthnitzer Street 40, 01187 Dresden, Germany; ‡ Department of Physics and Astronomy, University of California, Irvine, California 92697, United States

## Abstract

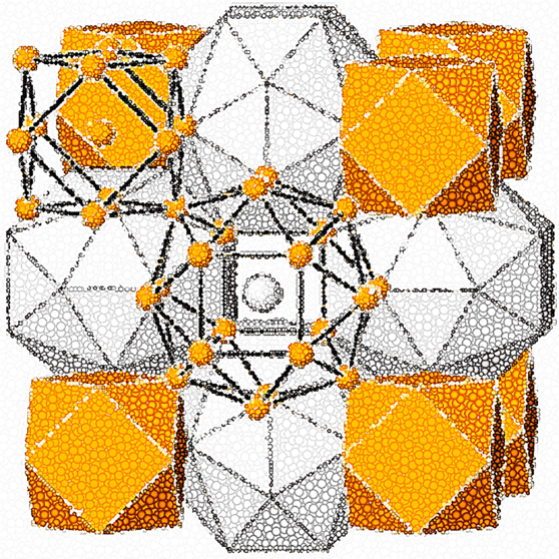

In the phase diagram U-Cd, only one compound has been identified so far—UCd_11_ (space group *Pm*3̅*m*). Since the discovery of this material, the physical properties of UCd_11_ have attracted a considerable amount of attention. In particular, its complex magnetic phase diagram—as a result of tuning with magnetic field or pressure—is not well-understood. From a chemical perspective, a range of lattice parameter values have been reported, suggesting a possibility of a considerable homogeneity range, *i.e.*, UCd_11–x_. In this work, we perform a simultaneous study of crystallographic features coupled with measurements of physical properties. This work sheds light on the delicate relationship between the intrinsic crystal chemistry and magnetic properties of UCd_11_.

## Abstract 2

A comprehensive study of crystallographic and physical properties of UCd_11_ has been carried out to evaluate the relationship between intrinsic crystal chemistry and magnetism of this material. It was found that this material shows a very narrow homogeneity range. Additionally, the antiferromagnetic ground state appears to remain the same across several studied samples.

## 1 Introduction

The initial interest in the compounds of cadmium and uranium was motivated by the possible application of these materials in reprocessing of nuclear fuels.^1,2^ Within the uranium–cadmium system, only one compound has been reported so far—denoted as UCd_11_ (structure type BaCd_11_).^1−8^ Its detailed structure determination using neutron diffraction revealed strong cadmium deficiency, distributed more or less equally over all occupied Cd sites. As a result, the cadmium-deficient composition of UCd_9.5_ was reported.^12^ Measurements of magnetic properties of UCd_11_ revealed that this compound orders antiferromagnetically below *T*_N_ = 5 K.^9−11^ However, what has really spiked scientific interest in this material was an enhancement of the effective electron mass, as evidenced by one of the largest electronic specific heat coefficients γ among known uranium-based materials.^12,9^ From the specific heat data, γ of approximately 950 mJ mol^–1^ K^–2^ has been estimated.^9,13,14^ However, de Haas–van Alphen experiments performed later^15^ have suggested that such a large value of γ is likely caused by the magnetic specific heat rather than by heavy quasiparticles. Based on the de Haas–van Alphen measurements, γ in the antiferromagnetic state was estimated to be approximately 250 mJ mol^–1^ K^–2^.^15^

The uranium–uranium distance in UCd_11_ is rather large (*d*_U–U_ = 6.56 Å), much larger than the Hill limit (*d*_U–U_ = 3.5 Å)^16^—this is consistent with the localized nature of *f*-orbitals in this material.^17−19^ However, the magnetic phase diagram of UCd_11_ appears to be rather complex: in addition to the antiferromagnetic transition, another weaker transition is observed around *T* = 2 K.^20,21^ Moreover, upon application of modest hydrostatic pressure (*p* ≤ 20 kbar), two additional transitions appear below the ambient-pressure antiferromagnetic one.^22,23^ Similarly, several metamagnetic transitions have also been observed.^20,21,24^ The nature of all of these transitions remains unclear. Overall, while μSR spectroscopy and neutron diffraction experiments on UCd_11_ confirmed bulk magnetic ordering below *T*_N_,^12,25^ the properties of this material appear to be rather intricate and remain to be understood completely.^10,12,13,18,21,26^

**Figure 1:**
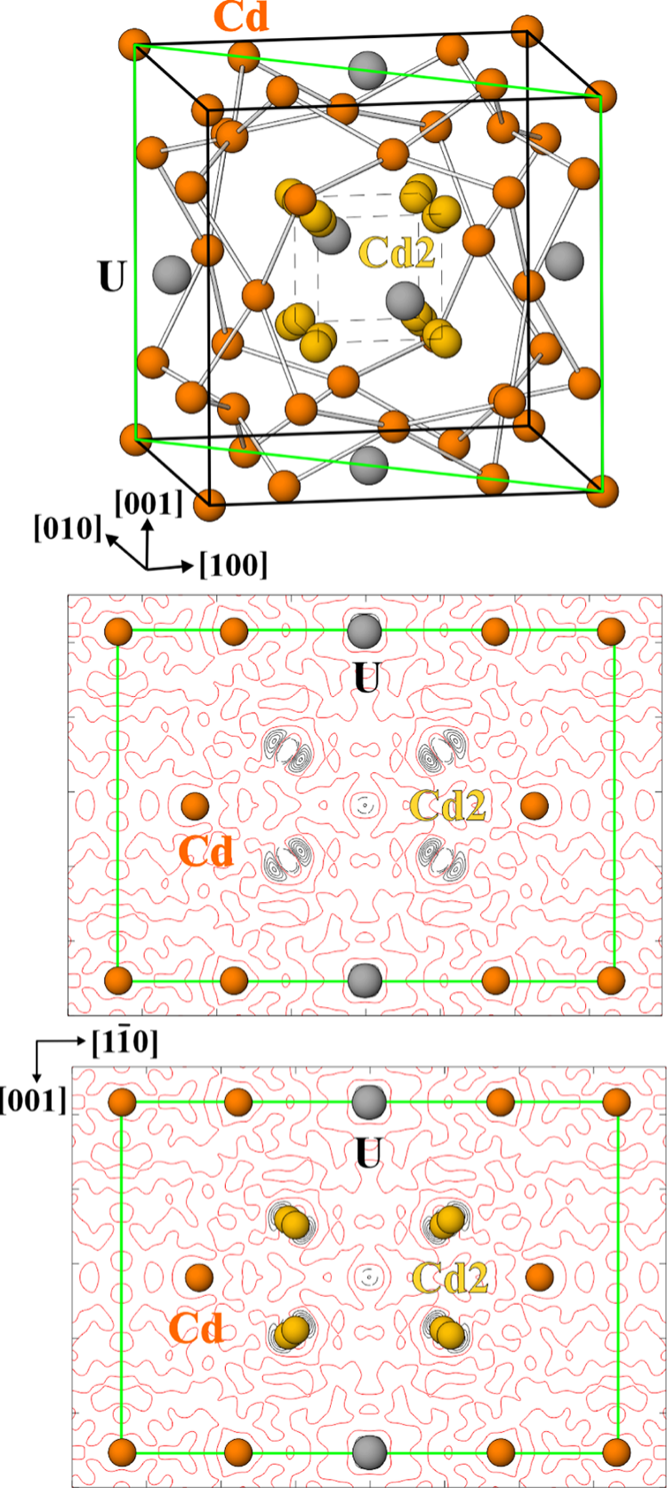
Crystal structure determination of UCd_11_: (top) unit cell along *ca.* [010]; (middle) difference electron density map in the diagonal plane (green), calculated without Cd2 site, the atoms used for the calculation are shown; (bottom) difference electron density map in the (110) plane decorated with all observed atoms. Red lines—zero level, black solid and black dashed lines denote positive and negative levels, respectively.

**Figure 2:**
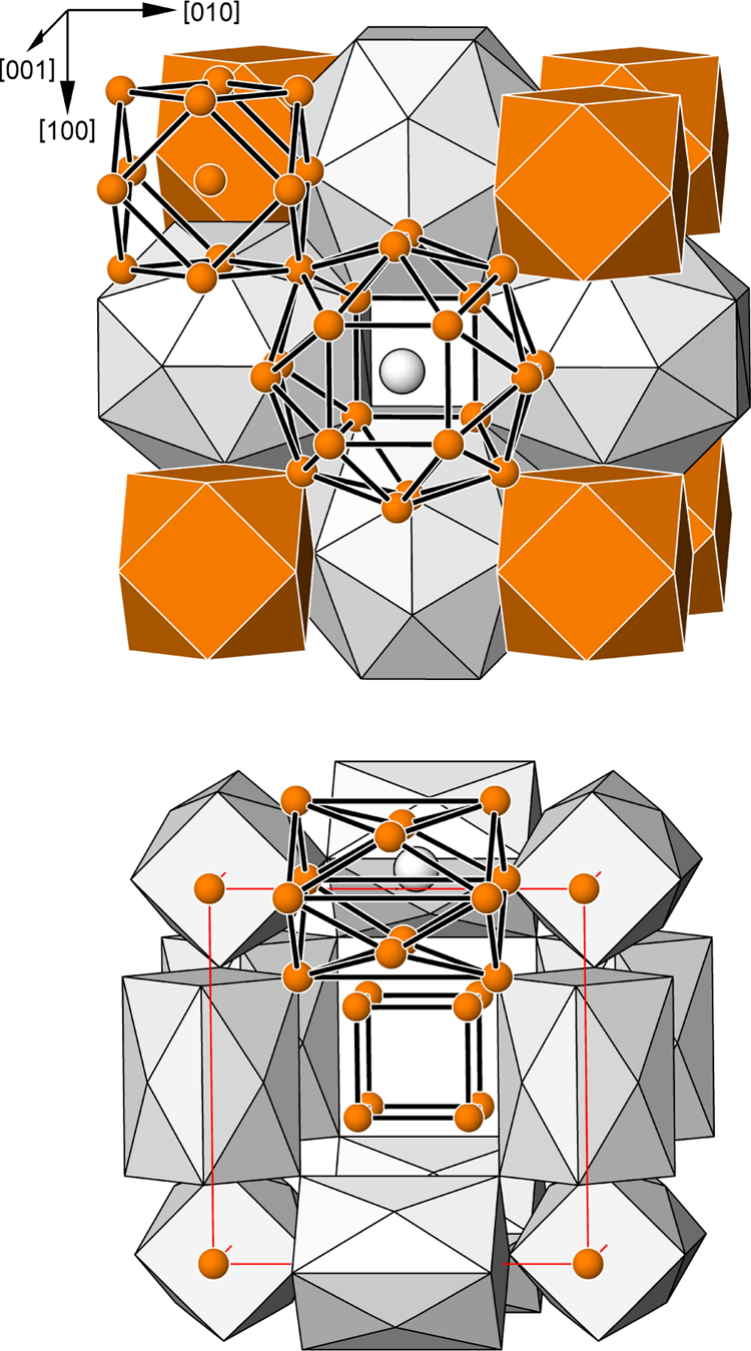
(top) Crystal structure of UCd_11_ represented as an arrangement of cubooctahedra around Cd1 position (orange) and uranium-centered polyhedra (gray) formed by 20 Cd atoms. (bottom) Cavities in the structure of UCd_11_: cubes around (^1^/_2_^1^/_2_^1^/_2_) site and four-capped tetragonal prisms around the (^1^/_2_ 0 0) site.

Atomic arrangement in UCd_11_, assuming complete occupancy of all Cd sites, is fairly complex, hosting uranium-centered polyhedra coordinated by 20 cadmium atoms, see Figure 1. Four cadmium sites and one uranium site exist in UCd_11_.^12^ It is likely that the origin of peculiar magnetism in UCd_11_ stems from its complex crystal structure (Figures 2). A large span of lattice parameter values is reported in the literature (9.248 Å ≤ *a* ≤ 9.29 Å), suggesting a possible homogeneity range as a result of partial occupancy on the cadmium sites.^1,9,10,12−14,23^ Additionally, a variation of electronic specific heat coefficient γ from 803 to 950 mJ mol^–1^ K^–2^ has been observed.^9,13,14^ Similar to other uranium-based systems, a delicate interplay between intrinsic crystal chemistry and physical properties can only be revealed by a comprehensive analysis on both macro- and microscales.^27−35^

**Figure 3:**
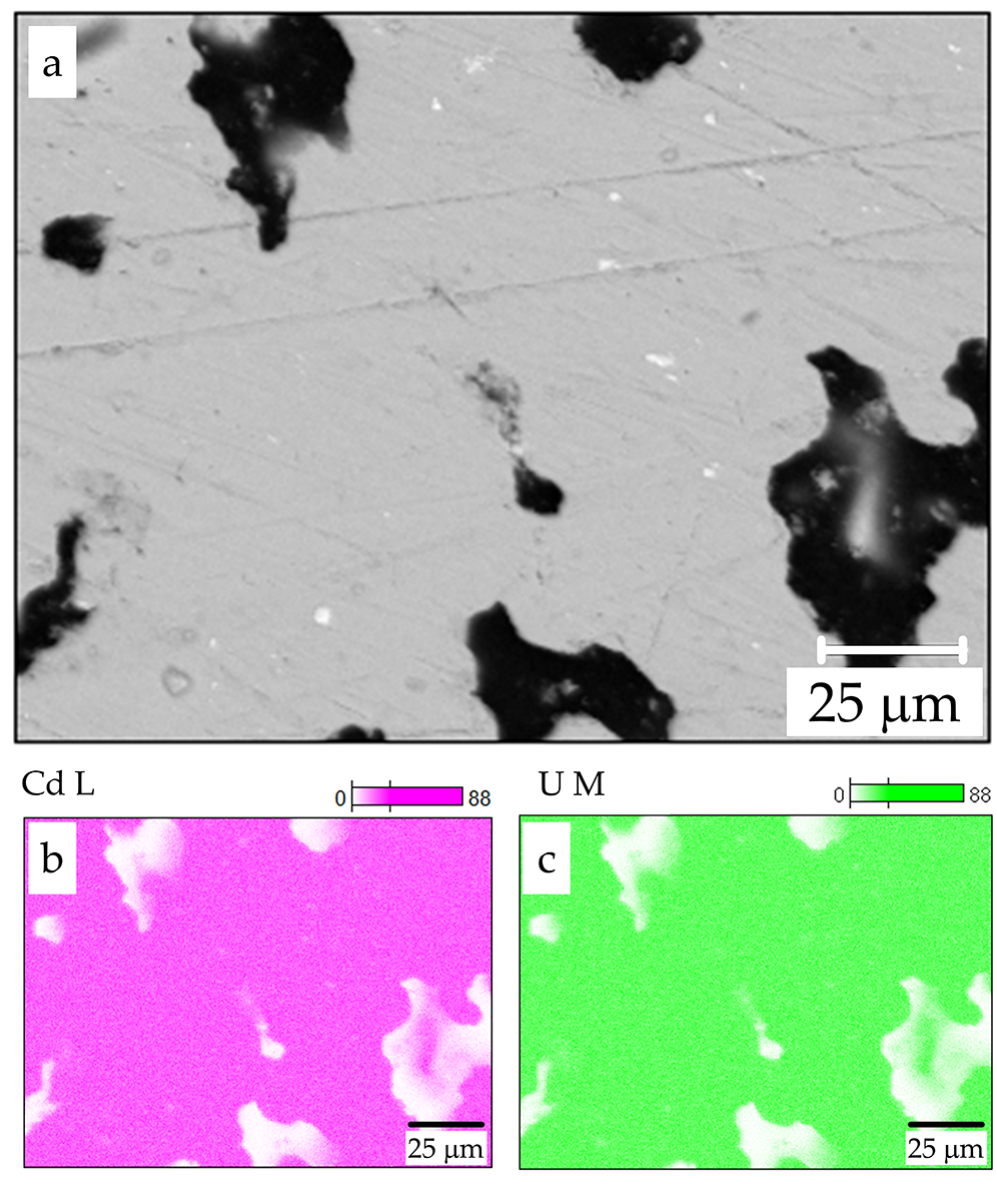
Backscatter electron micrograph of a polished UCd_11_ sample (sample 5). Panels (b) and (c) show elemental mappings for the same region, with Cd (pink) and U (green), respectively. No distinguishable variation in the sample composition has been observed.

**Figure 4:**
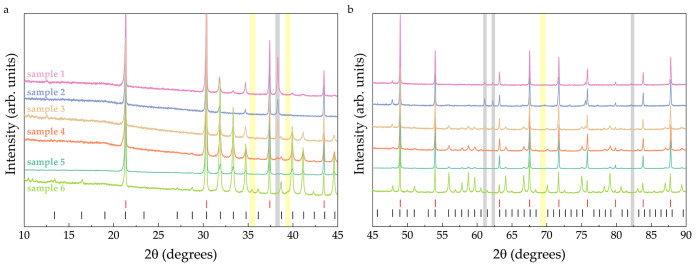
Powder X-ray diffraction data for the UCd_11_ samples used in the current study. Black vertical symbols mark the positions of the reflections corresponding to the UCd_11_ phase, while red symbols correspond to LaB_6_ used as a standard. The gray and yellow regions mark the peak positions of elemental Cd and elemental U, respectively. For these, only the peaks which do not overlap with either UCd_11_ or LaB_6_ are shown.

**Figure 5:**
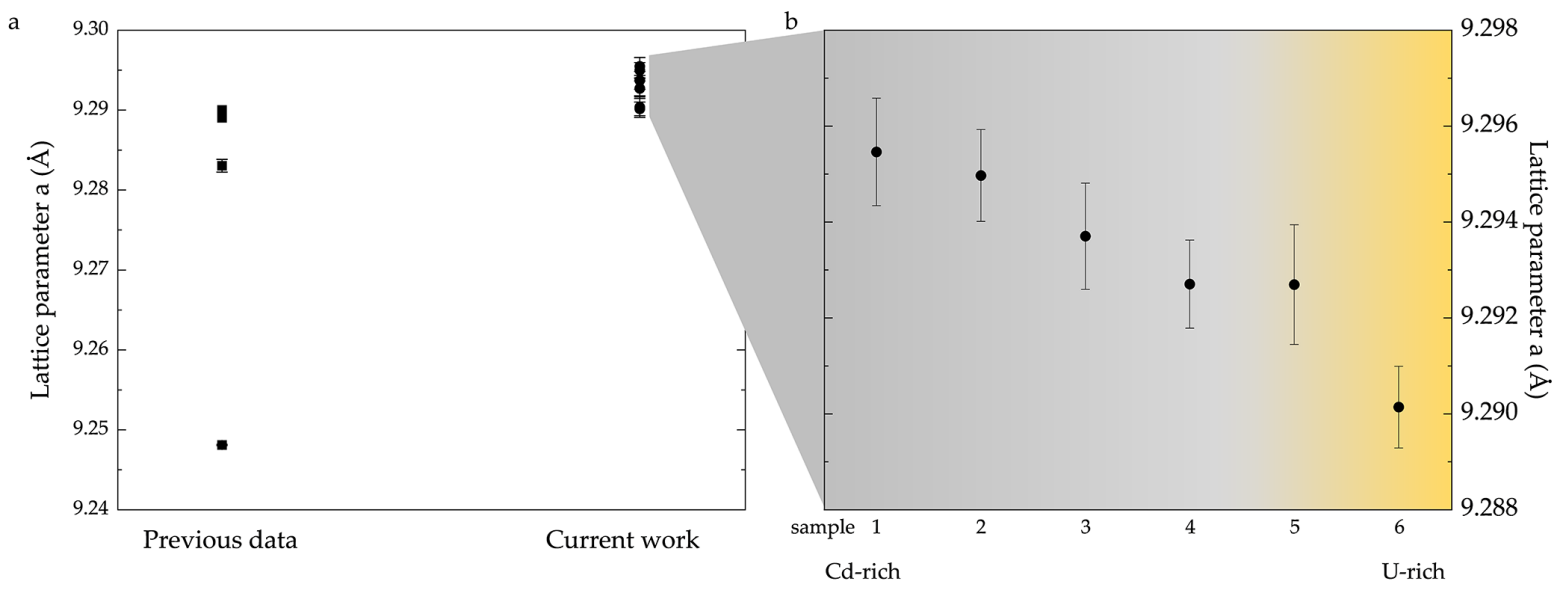
(a) Summary of lattice parameters for UCd_11_—the previously reported values are shown as squares,^1,9,10,12−14,23^ while the results of the present investigations are shown as circles. (b) Enlarged view of the region containing lattice parameter values of UCd_11_ from the current study. The shown error bars are the standard deviation multiplied by a factor of three. While it is not possible to determine the exact amount of elemental Cd or U within our samples, the relative amount is estimated from the powder X-ray data (see the Methods section for details).

## 2 Methods

### 2.1 Synthesis

Six U-Cd samples with three U:Cd ratios (6.3:93.7, 8.3:91.7, and 10.3:89.7, see Table S1) were prepared by direct reaction of the components. All sample preparation and handling were performed in the specialized laboratory equipped with an argon-filled glovebox system (MBraun, *p*(H_2_O/O_2_) < 0.1 ppm).^36^ Similar to previous studies,^8,10^ single crystals of UCd_11_ were obtained by melting (i) stoichiometric and (ii) slightly off-stoichiometric amounts of uranium (powder prepared from sheet, Goodfellow, 99.98%) and cadmium (pieces, Alfa Aesar, >99.9%). The tantalum tubes with the starting mixture of the elements were sealed under an argon atmosphere, heated to 420 °C, and then slowly cooled back to room temperature at a rate of 2.5 °C per hour. The resultant product was a gray, polycrystalline powder with some μm-sized crystals.

### 2.2 Materials Characterization

Powder X-ray diffraction was performed on a Huber G670 imaging-plate Guinier camera with a Ge-monochromator (CuK_α1_, λ = 1.54056 Å). LaB_6_ was used as a standard. Phase identification was done using WinXPow software.^37^ For all samples, in addition to the main UCd_11_ phase which is homogeneous (Figure 3), a small amount of either elemental Cd (samples 1–5) or elemental U (sample 6) is seen in the powder X-ray diffraction data (see Figure 4 and Table S2). While it is not possible to estimate the exact amount of Cd or U admixture, the relative amounts (compared to other samples in the study) were found from the ratio of the intensities of the strongest UCd_11_ (2θ = 31.9 degrees) to the strongest Cd (2θ = 38.3 degrees) peaks. The lattice parameters were determined by a least-squares refinement using the peak positions extracted by profile fitting (WinCSD software^38^, Figure 5).

Small crystals on the order of *ca.* 30 μm were suitable for single-crystal diffraction experiments. The diffraction data were collected using a Rigaku AFC7 diffractometer equipped with a Saturn 724+ CCD detector and a MoK_α_ radiation source (λ = 0.71073 Å). The WinCSD^38^ software packages were used for data analysis. The results of the crystallographic characterization of UCd_11_ are provided in Tables 1–3 and S2. From the structural refinement, no variation in stoichiometry or occupancy was observed between various UCd_11_ samples. Thus, all samples’ stoichiometry is UCd_11_ within experimental error bars.

**Table 1:** Crystallographic Data for UCd_11_

composition	UCd_11_
space group	*Pm*3̅*m*
Pearson symbol	*cP*36
formula units per unit cell, *Z*	3
lattice parameters	
*a*/Å	9.2970(3)
*V*/Å^3^	803.58(8)
calc. density/g cm^–1^	9.14
crystal form*	irregular shaped
crystal size/μm	12 × 12 × 30
diffraction system	RIGAKU AFC7
detector	Saturn 724+ CCD
radiation, λ/Å	MoKα, 0.71073
scan; step/degree; *N*(images)	φ, 0.6, 1200
maximal 2θ/degree	82.0
range in *h*, *k*, *l*	–17 ≤ *h* ≤ 16
	–17 ≤ *k* ≤ 16
	–14 ≤ *l* ≤ 6
absorption correction	multi-scan
*T*(max)/*T*(min)	0.661
absorption coeff./mm^–1^	36.3
*N*(*hkl*) measured	15 983
*N*(*hkl*) unique	590
*R*_int_	0.052
*N*(*hkl*) observed	579
observation criteria	*F*(*hkl*) ≥ 4σ(*F*)
refined parameters	16
*R*_F_	0.033
*R*_w_	0.037
residual peaks/e Å^–3^	–0.64/1.05

For metallographic investigations, pieces of UCd_11_ samples were embedded into a polymer matrix using a hot mounting press (ProntoPress 10). SiC paper and diamond powder with grain sizes of 3 μm or smaller were used for surface polishing. Chemical composition was studied on polished samples using energy-dispersive X-ray spectroscopy with a Jeol JSM 6610 scanning electron microscope equipped with an UltraDry EDS detector (Thermo Fisher NSS7). The semiquantitative analysis was performed with 25 keV acceleration voltage and ≈3 nA beam current. The resultant backscatter electron micrograph is shown in Figure 3. Aside from cavities (black), no inclusions of secondary phases have been detected. Elemental mapping of Cd (panel (b)) and U (panel (c)) indicates that the uranium content remains constant within the sample (8.5 at. % of U).

### 2.3 Physical Property Measurements

The analysis of magnetism in UCd_11_ was performed on polycrystalline samples. For most of the samples, either elemental U or Cd was present in addition to the UCd_11_ phase (see Figure 4). The magnetic properties were studied using a Quantum Design (QD) Magnetic Property Measurement System for the temperature range from *T* = 1.8 K to *T* = 300 K and for applied magnetic fields up to *H* = 7 T. The specific heat data were collected on a Quantum Design Physical Property Measurement System in the temperature range from *T* = 0.4 K to *T* = 10 K for magnetic fields up to *H* = 9 T. Due to the polycrystalline powder-like nature of the samples, it was not possible to carry out electrical resistivity measurements.

## 3 Results and Discussion

The UCd_11_ compound crystallizes in the cubic BaHg_11_ structure type and decomposes peritectically at *T* = 473 °C.^1^ It was previously shown that the structure of UCd_11_ is rather robust against application of pressure—the unit cell can be compressed by nearly 20% (for *p* = 20 GPa) without any discernible structural phase transitions.^26^ While some structural information has been reported previously, the possibility of a homogeneity range in UCd_11_ has not been investigated.^1,9,10,12−14,23^

In the current study, high-quality single-crystal data was obtained for crystals extracted from samples 4 and 5. The collection of diffraction intensities was made on an irregular single crystalline specimen with dimensions of 12 × 12 × 30 μm^3^. Crystallographic information for the single-crystal UCd_11_ (sample 4), together with further details of the single-crystal X-ray diffraction experiment, are presented in Tables 1 and 2. The collected diffraction data were indexed using the cubic lattice with the lattice parameter of 9.2970(3) Å, being close to the upper limit of those reported earlier (9.248 Å ≤ *a* ≤ 9.29 Å).^1,9,10,12−14,23^ The extinction conditions in the measured data set agreed well with the primitive lattice of the BaHg_11_ structure type indicated in ref (12). Application of the charge-flipping technique allowed to establish the basic atomic arrangement formed by one uranium and four cadmium positions (Figure 1, top). It is important to note that the displacement parameter for the Cd2 position was strikingly large in comparison with three other cadmium sites.

**Table 2:** Atomic Coordinates and Isotropic/Equivalent Displacement Parameters (in Å^2^) in the Crystal Structure of UCd_11_

atom	site	*x*/*a*	*y*/*b*	*z*/*c*	U_eq/iso_^b^
U	3*c*	0	1/2	1/2	0.0146(2)
Cd1	1*a*	0	0	0	0.0152(3)
Cd2a^a^	8*g*	0.3352(2)	*x*	*x*	0.018(1)
Cd2b^a^	8*g*	0.3509(2)	*x*	*x*	0.018
Cd3	12*i*	0	0.23438(7)	*y*	0.0174(2)
Cd4	12*j*	1/2	0.15523(7)	*y*	0.0172(2)

a Occupancy of Cd2a was constrained with Cd2b (occ. Cd2a + occ. Cd2b = 1) and resulted in the occupancy ratio of Cd2a:Cd2b = 0.523(4):0.477. Both positions were refined using isotropic displacement parameters.

b U_eq_ = ^1^/_3_[U_11_a^*2^*a*^2^ + ··· 2U_23_b* c* *bc* cos α].

Refinement of this structure model using anisotropic approximation of atomic displacement revealed strong anisotropy of the atomic displacement for the Cd2 site. Calculation of the residual electronic density with isotropic displacement for this position indicates nondescribed density on both sides of the position along the space diagonal of the unit cell (Figure 1, middle), making a split necessary (Figure 1, bottom). The final refinement resulted in *R*_F_ of 0.033 for 579 reflections used. The attempts to resolve this split by lowering the symmetry within the same Laue class failed: despite the larger number of refined parameters, the residual values were not reduced (0.039 in the space group *P*4̅3*m* and 0.052 in *P*23), and the strong anisotropy of the Cd2 position remained unchanged. An attempt to implement the defect (approximately 14%, as suggested in ref (12)) on all Cd positions led to a significant increase of *R*_F_ to 0.056. Final atomic coordinates, displacement parameters, and interatomic distances are listed in Tables 2, 3, and S2, respectively.

**Table 3:** Anisotropic Displacement Parameters (in Å^2^) in the Crystal Structure of UCd_11_^a^

atom	U_11_	U_22_	U_33_	U_23_
U	0.0197(4)	0.0120(2)	U_22_	0
Cd1	0.0152(4)	U_11_	U_11_	0
Cd3	0.0180(3)	0.0168(2)	U_22_	0.0016(3)
Cd4	0.0172(3)*i*	0.0175(2)	U_22_	0.0018(3)

a U_12_ = U_13_ for all positions.

The crystal structures of the BaHg_11_ structure type are usually described as a packing of the large cation-centered polyhedrons (UCd_20_ in the case of UCd_11_) with 20 vertices located at (0 ^1^/_2_^1^/_2_) and Cd-centered cuboctahedrons (CdCd_12_) at (0 0 0)—see, for example, Figure 2 (top panel) in refs (39) and (40). This description does not allow us to understand the reasons for the split of the Cd2 site. Besides filled cages in the Cd framework, two types of smaller empty voids exist: in the nonsplit model, a cubic one is located at (^1^/_2_^1^/_2_^1^/_2_) and the four-capped tetragonal prismatic one at (^1^/_2_ 0 0) (see Figure 2, bottom panel); in the split model, both are distorted. As it was recently shown for several structures, empty cubic voids in the frameworks are not stable and undergo deformation either toward tetragonal antiprisms or toward tetrahedron stars like in Ce_2_Ga_12_Pt,^41^*R*_2_Ga_2_*T*,^42^ Ce_2_PdGa_12_,^43^ and PuGa_6_.^44^ The split of Cd2 position in UCd_11_ can be understood as a result of the structural transformation according to the latter scenario. Similar strong displacement anisotropy was observed in the ternary derivative YbPd_3_Ga_8_^45^ but not in the prototype BaHg_11_.^40^

The strong displacement anisotropy of the Cd2 position may have two origins: the dynamic and the static one. Independently of the origin, the Cd2 position or its split positions is 3.8 Å apart from the U one. Thus, this should not significantly influence the electronic state of U and, therefore, its magnetic moment. On the other hand, if the Cd positions located in the first coordination sphere of U are not fully occupied, as proposed by ref (12), the magnetic properties of uranium may change. The comparison of the magnetic susceptibility measurements of current and previous works reveals the same ordering temperature and effective magnetic moment (see below), which indicates that the electronic state of uranium is in fact the same across all UCd_11±x_ samples reported so far.

Partial occupancy on the Cd sites has been previously suggested, resulting in UCd_9.5_ composition.^12^ It is important to note that the analysis of the crystal structure of UCd_11_ in ref (12) was carried out using neutron powder diffraction experiments. The sample investigated in ref (12) shows the lattice parameter on the lower end of the spectrum of known values (Figure 5a). It was not possible to reproduce this value of the lattice parameter in the current study—the single-crystal investigations yielded larger lattice parameter values. It is possible, however, that the defects on Cd sites reported in ref (12) may appear due to the thermal treatment during the single-crystal growth.

**Figure 6:**
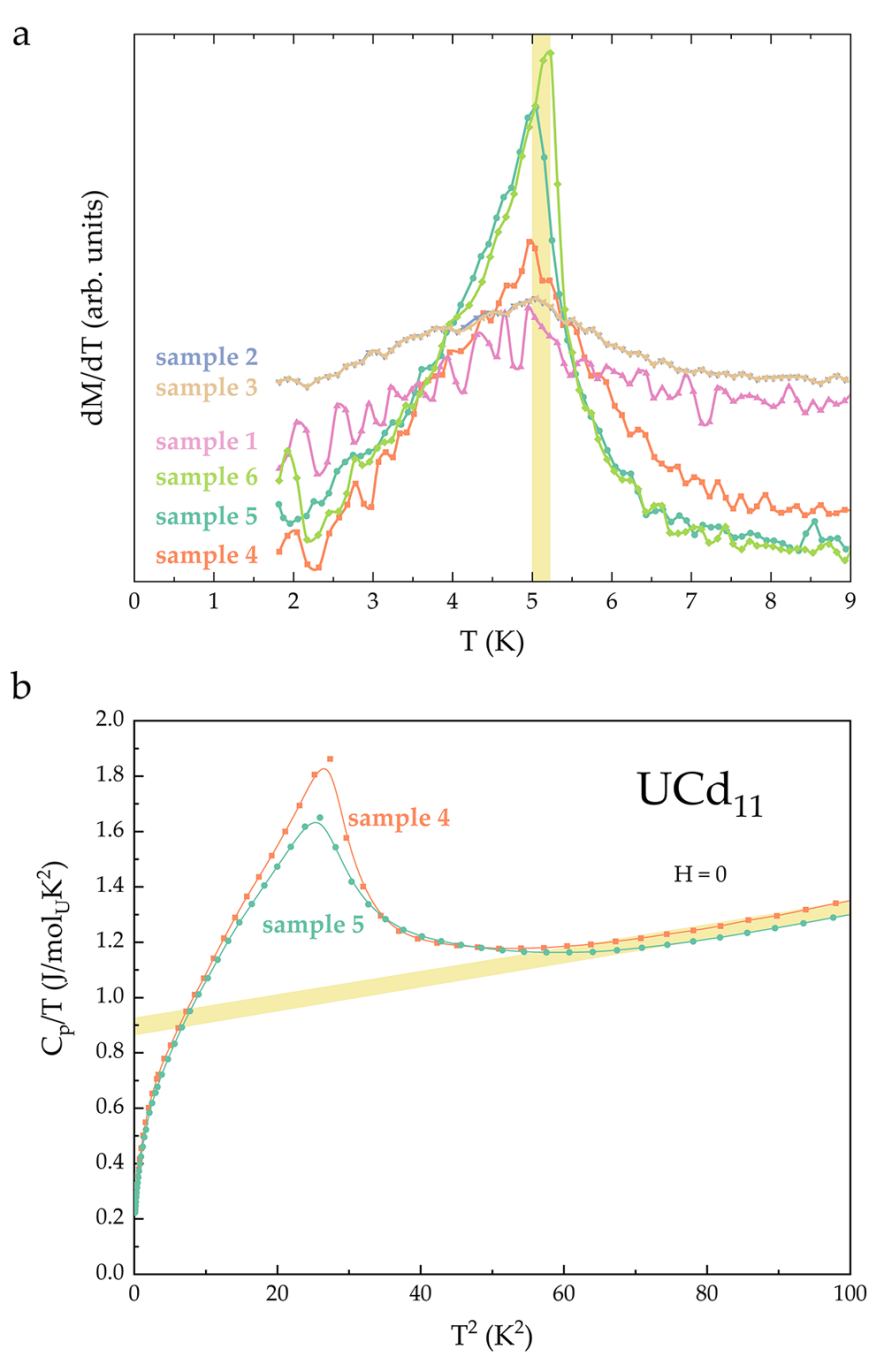
(a) Derivative of the temperature-dependent magnetization data for UCd_11_. The yellow region highlights the range of the values of the ordering temperature *T*_N_. (b) Specific heat of UCd_11_, scaled by temperature, as a function of temperature squared for two of the samples from the current study. The yellow region highlights the range for the values of the electronic specific heat coefficient γ.

Magnetic properties of UCd_11_ have been previously studied using a number of experimental techniques.^9−11^ The entrance into antiferromagnetic state is marked by a characteristic anomaly around *T*_N_ = 5 K. For all samples from the current study, a cusp-like feature has been observed in temperature-dependent magnetization data at *T* = 5.1 ± 0.1 K, as evidenced by the derivative d*M*/d*T*, shown in Figure 6a (see also Table S1). Assuming that with a larger lattice parameter, the composition is the stoichiometric one and the structural deformation takes place in the cube-like arrangement in the middle of the unit cell, the low lattice parameter can produce defects in the cadmium sub-lattice. The resultant structural deformation does not influence the magnetic behavior, since the affected part of the Cd framework is located far from the uranium atoms (∼3.8 Å). Magnetism of UCd_11_ is likely mediated by the RKKY interactions, which means that the magnetic susceptibility is not influenced by the crystallographic point defects. However, the cubic symmetry of the uranium coordination is locally violated, which leads to reduced peak sharpness in the d*M*/d*T* data. The inverse susceptibility data were fit to the Curie–Weiss law. In agreement with previous work,^9^ an effective moment μ_eff_ = 3.43 μ_B_ F.U.^–1^ and Weiss temperature θ_W_ = −23 K were established.

The antiferromagnetic ordering of UCd_11_ is also marked by an anomaly in the specific heat data, as shown in Figure 6b. The sharpness of this feature is somewhat reduced compared to some of the previous reports, which can likely be attributed to the polycrystalline nature of the samples. The height and position of the jump are comparable to previous studies.^9,13,14,21^ In the normal state, the specific heat data, scaled by temperature, can be fitted with a linear relation, yielding the value of the Sommerfeld coefficient γ. For the present study, the range of γ values 860 mJ mol^–1^ K^–2^ ≤ γ ≤ 920 mJ mol^–1^ K^–2^ is consistent with previous reports.

## 4 Conclusions

In the current work, we present a detailed study of crystallographic properties of UCd_11_. We have prepared and characterized a series of UCd_11_ samples grown from both uranium-rich and cadmium-rich sides of the binary phase diagram. Given that only one uranium–cadmium compound has been reported so far, all of the studied samples contain either elemental cadmium or elemental uranium in addition to the UCd_11_ phase (see Figure 5b). Contrary to the literature data, we find a rather small span of the lattice parameter values. This suggests that the homogeneity range of UCd_11_ is likely narrow. Additionally, the occupancy of the uranium-centered polyhedra appears to be complete, contrary to the previous report.^12^ The value of the electronic specific heat coefficient γ for UCd_11_, extracted from the specific heat data above the antiferromagnetic transition, γ = 890 ± 30 mJ mol^–1^ K^–2^ is similar to previous studies. The antiferromagnetic ordering temperature *T*_N_ = 5 K does not appear to vary between different UCd_11_ samples. This indicates that magnetism in UCd_11_ is likely rather robust to small changes in crystal features, which is typically not the case for strongly correlated actinide- and lanthanide-based materials.

## Supporting Information Available

Crystallographic data are provided in the supporting information (PDF)
