# Supplementary material for: Crystal Chemistry and Physics of UCd11
Source: Inorg Chem. 2022 Nov 29;61(49):19695–701. doi: 10.1021/acs.inorgchem.2c01986 (PMC9749020; doi:10.1021/acs.inorgchem.2c01986)
Supplement: Supplementary file 1 — ic2c01986_si_001.pdf [file ic2c01986_si_001.pdf]

# Crystal chemistry and physics of $UCd_{11}$

## Supporting Information

Nazar Zaremba,[1] Kristian Witthaut,[1] Yurii Prots,[1] Mitja Krnel,[1] Ulrich Burkhardt,[1] Zachary Fisk,[2] Yuri Grin,[1] and Eteri Svanidze[1,*]

[1] Department Chemische Metallkunde, Max-Planck-Institut für Chemische Physik fester Stoffe, Nöthnitzer Str. 40, 01187 Dresden (Germany)

[2] Department of Physics and Astronomy, University of California, Irvine, California, 92697 (USA)

[*] E-Mail: svanidze@cpfs.mpg.de

***Table S1:*** *Summary of prepared $UCd_X$ samples*

| Sample number | Starting U:Cd ratio (atomic) | Phases identified in powder X-ray data | Lattice parameter, 3σ (Å) | Ordering temperature (K) |
|---|---|---|---|---|
| 1 | 10.3:89.7 | $UCd_{11}$ + Cd | 9.29546 ± 0.00112 | 5.0 ± 0.1 |
| 2 | 6.3:93.7 | $UCd_{11}$ + Cd | 9.29496 ± 0.00096 | 5.1 ± 0.1 |
| 3 | 6.3:93.7 | $UCd_{11}$ + Cd | 9.29369 ± 0.00111 | 5.1 ± 0.1 |
| 4 | 8.3:91.7 | $UCd_{11}$ + Cd | 9.29269 ± 0.00092 | 5.0 ± 0.1 |
| 5 | 8.3:91.7 | $UCd_{11}$ + Cd | 9.29268 ± 0.00125 | 5.0 ± 0.1 |
| 6 | 10.3:89.7 | $UCd_{11}$ + U | 9.29012 ± 0.00086 | 5.2 ± 0.1 |

***Table S2:*** *Interatomic distances in the crystal structure of $UCd_{11}$ (sample 4).*

| Atoms | | *d*, Å | Atoms | | *d*, Å | Atoms | | *d*, Å |
|---|---|---|---|---|---|---|---|---|
| U | 4Cd3 | 3.5299(6) | Cd2b | 3Cd4 | 2.849(1) | Cd4 | 2Cd3 | 2.849(1) |
| | 8Cd4 | 3.5530(7) | | 3 { Cd2a* | 2.957(2) | | 2Cd4 | 2.9174(9) |
| | 8Cd2b* | 3.836(1) | | Cd2b* } | 3.098(2) | | 2 { Cd2a* | 2.954(1) |
| | 8Cd2a* | 3.847(1) | | 3Cd3 | 3.422(1) | | Cd2b* } | 2.849(1) |
| Cd1 | 12Cd3 | 3.1148(6) | | 3U | 3.836(1) | | 4Cd3 | 2.9852(7) |
| Cd2a | 3 { Cd2a* | 2.802(2) | Cd3 | 4Cd4 | 2.9852(7) | | 2U | 3.5530(7) |
| | Cd2b* } | 2.957(2) | | 1Cd1 | 3.1148(6) | | | |
| | 3Cd4 | 2.954(1) | | 4Cd3 | 3.1148(6) | | | |
| | 3Cd3 | 3.643(1) | | 2 { Cd2b* | 3.422(1) | | | |
| | 3U | 3.847(1) | | Cd2a* } | 3.643(1) | | | |
| | | | | 1U | 3.5299(6) | | | |

*Positions Cd2a and Cd2b are partially occupied: occ(Cd2a) : occ(Cd2b) = 0.523(4) : 0.477. Only one position can be occupied.
